# Acute Hematogenous Periprosthetic Hip Infection by *Gemella morbillorum*, Successfully Treated with Debridement, Antibiotics and Implant Retention: A Case Report and Literature Review of Osteoarticular *Gemella morbillorum* Infections

**DOI:** 10.3390/tropicalmed7080191

**Published:** 2022-08-18

**Authors:** Albert Pardo-Pol, Daniel Pérez-Prieto, Albert Alier, Lucas Ilzarbe, Lluïsa Sorlí, Lluis Puig, Santos Martínez-Díaz, Joan Gómez-Junyent

**Affiliations:** 1 Department of Orthopedics, Hospital del Mar, Institut Hospital del Mar d’Investigacions Mèdiques (IMIM), Universitat Autònoma de Barcelona (UAB), CEXS-Universitat Pompeu Fabra, 08003 Barcelona, Spain; 2 Department of Gastroenterology, Hospital del Mar, Institut Hospital del Mar d’Investigacions Mèdiques (IMIM), Universitat Autònoma de Barcelona (UAB), CEXS-Universitat Pompeu Fabra, 08003 Barcelona, Spain; 3 Department of Infectious Diseases, Hospital del Mar, Infectious Pathology and Antimicrobial Research Group (IPAR), Institut Hospital del Mar d’Investigacions Mèdiques (IMIM), Universitat Autònoma de Barcelona (UAB), CEXS-Universitat Pompeu Fabra, 08003 Barcelona, Spain

**Keywords:** prosthetic joint infection, *Gemella morbillorum*, intestinal bacterial translocation, implant retention

## Abstract

*Gemella morbillorum* is a facultative anaerobic, catalase-negative and non-spore forming Gram-positive cocci. It can be found as part of the normal oropharyngeal flora, in the gastrointestinal tract and the female genital tract. However, it can be a causal agent of infections such as endocarditis, meningitis or brain abscesses, and very rarely can cause osteoarticular infections. Herein, a case report of an acute hematogenous prosthetic hip infection caused by *Gemella morbillorum*, successfully treated with a DAIR and beta-lactam antibiotic therapy, is presented. We provide a literature review of the other orthopedic-related infections caused by this microorganism.

## 1. Introduction

Periprosthetic joint infection (PJI) is a serious complication that can occur after arthroplasty. It causes significant patient morbidity and poses a significant cost-burden for the healthcare system. It occurs in 1 to 2% of primary arthroplasties, increasing to 4% in revision surgeries [1,2]

The prostheses can become infected in three ways. Infection can occur perioperatively, mostly through intraoperative inoculation, or after an hematogenous infection, occurring at any time after implantation. It can also be the result of something more direct, such as spreading from a nearby infection e.g., adjacent osteomyelitis or soft tissue infection [3].

*Gemellamorbillorum* (formerly known as *Streptococcus morbillorum*) is a facultative anaerobic, catalase-negative and non-spore forming Gram-positive cocci [4]. It can be found as part of the normal oropharyngeal flora, in the gastrointestinal tract and the female genital tract. However, it can be a causal agent of infections such as endocarditis, meningitis or brain abscesses [5]. *G. morbillorum* was first described by Tunnicliff in 1917 [4]. Based on its biomolecular characteristics, it was included in the *Gemella* family in 1988. It has previously been denominated as *Diplococcus morbillorum, Peptococcus morbillorum, Peptostreptococcus morbillorum* and *Streptococcus morbillorum*. Currently, there are four recognized species of *Gemella spp.: G. haemolysans, G. morbillorum, G. sanguinis* and *G. bergeriae* [6]. Dental procedures, gastrointestinal carcinomas [7], hepatorenal diseases, diabetes mellitus and steroid therapy are predisposing factors of infections in humans [8,9].

Microbiological identification may be difficult, and the infection can go undetected. Therefore, its true incidence has not been confirmed [10,11]. *Streptococcus viridians* may be misleading in microbiological studies because it also presents with alpha-hemolysis in the blood agar. Sometimes, a polymerase chain reaction (PCR) may be needed for the proper identification of *Gemella* spp. infections, such as bacteremia, endocarditis or those affecting the central nervous system [10,11,12].

Infections by *G. morbillorum* have been identified in all age groups and in both immunocompetent and immunosuppressed patients. However, anecdotal case reports of osteoarticular infections caused by *G. morbillorum* have been reported [13,14,15,16,17,18,19].

Herein, a case report of an acute hematogenous prosthetic hip infection caused by *G. morbillorum* is presented. A literature review of the other orthopedic-related infections caused by this microorganism is also provided.

## 2. Case Report

This case involves a 60-year-old woman with a history of severe psoriasis being treated with Risankizumab, a humanized immunoglobulin G1 (IgG1) monoclonal antibody selective to the interleukin (IL)-23 protein.

**Figure 1:**
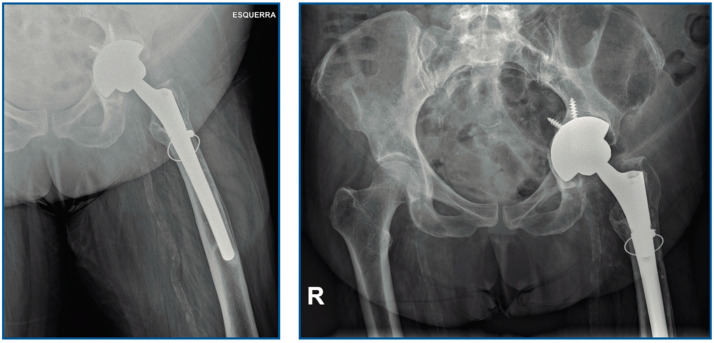
Hip radiography showing the implanted prosthesis.

Her past medical history included obesity (body mass index 37 kg/m^2^), major depressive disorder (MDD) and chronic alcohol abuse. In 2011, a cemented total hip arthroplasty (THA) U2 prosthesis (United Orthopedic Corporation, New Taipei, Taiwan) was implanted after the woman experienced a sub-capital fracture. In 2016, a one-step prosthetic exchange was performed due to stem loosening, for which a revision prosthesis (Zimmer Biomet, Wayne Township, IN, USA) was implanted (Figure 1). PJI was ruled out based on the IDSA’s 2013 criteria [20].

In February 2021, the patient came to the emergency department with acute pain in the operated hip, fever and erythema in the wound, which led us to suspect an acute periprosthetic infection. The blood test showed leukocytosis (10.2 × 10^9^/L) and an elevated level of C-reactive proteins (CRP: 153.6 mg/L). Arthrocentesis was performed and a PJI was diagnosed (231,000 cells/mm, of which 91% were neutrophils) according to the EBJIS criteria [21].

On the same day of admission, the patient underwent DAIR (debridement, antibiotics, irrigation and implant retention). A thorough debridement was performed to remove all avascular and/or necrotic tissue. An exchange of the acetabular liner and the femoral head was performed. The removed components were sent for sonication, along with five periprosthetic tissue samples for microbiological analysis. A histopathological analysis was requested for two samples. Non-alcoholic chlorhexidine irrigation with 9 liters was performed. The wound was closed, and incisional negative-pressure therapy (PICO) was carried out, along with two deep drains. Due to acute renal failure (GFR 41 mL/min 1.73 m^2^; 1.62 mg/dL creatinine), vancomycin was avoided and an empirical broad-spectrum antibiotic therapy with intravenous daptomycin 850 mg/daily (10 mg/kg/day of actual body weight) and ceftazidime 2 g/12 h, administered by intermittent infusion, was initiated.

**Figure 2:**
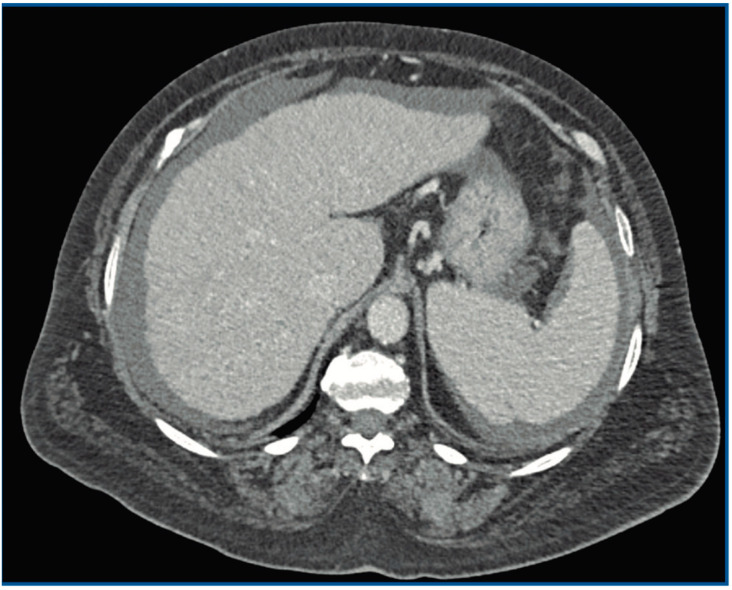
CT scan showing chronic inflammation of the liver with moderate ascites.

The intraoperative tissue, synovial fluid and sonication fluid cultures were positive for *G. morbillorum* (susceptible to penicillin and cefotaxime). After those results, antibiotic therapy was modified to entail the intermittent infusion of 2 g cefotaxime every 8 h, in monotherapy. An echocardiography showed no vegetations or other signs of endocarditis. An abdominal CT scan ruled out malignancy, but the liver parenchyma showed signs of a previously undiagnosed chronic inflammation with moderate ascites (Figure 2). The gastroscopy showed signs of mild portal hypertension (without esophageal varices). During the colonoscopy, a sessile adenoma was removed. The patient also denied a history of dental procedures over the past few months.

We suspected a mixed etiology of cirrhosis (a combination of heavy alcohol intake and nonalcoholic fatty liver disease). The laboratory results showed a GGT 411 U/L and ALP 151 U/L, hypoalbuminemia (1.7 g/dL), an elevated AST (54 U/L) with normal ALT (20 U/L), cholesterol of 90 mg/dL and TG 64 mg/dL. The patient had a high MCV (111.3 fL) and elevated gamma globulins (IgG 2308 mg/dL, IgA 1212 mg/dL and IgM 202 mg/dL), but with a negative autoimmunity study (ANA, AMA and SMA). HBV, HCV and HIV serologies were negative.

In conclusion, the patient was diagnosed with an acute hematogenous PJI after spontaneous bacteremia due to gastrointestinal bacterial translocation, and in the context of decompensated cirrhosis (possibly of mixed etiology, due to excessive alcohol intake and nonalcoholic fatty liver disease).

The liver pathology revealed significant alterations in coagulation and severe hypoproteinemia (albumin levels 1.7–2 g/dL). These factors may have contributed to the persistent wound discharge. Due to this persistent drainage, despite the culture-negative swabs, a second DAIR was performed 10 days after the first surgery, with all cultures being negative in this case. At two weeks after the second intervention, the patient was discharged. Antibiotic therapy was modified to 1 g of oral amoxicillin every 8 h.

The patient went on to complete 3 months of uneventful antibiotic treatment. Although the patient was at risk of experiencing sporadic infections (due to her immunosuppressive treatments for psoriasis and cirrhosis), we consider the infection cured, so we decided not to use suppressive treatment.

At the 18-month follow-up appointment, the patient was pain-free, with a Postel-Merle-d’Aubigné of 6-6-6 and a current BMI of 27 Kg/m^2^. The liver cirrhosis is currently being monitored by hepatology and no further decompensation has presented.

## 3. Discussion

We present a case of an acute hematogenous PJI caused by *G. morbillorum* that manifested with acute pain, functional impotence and fever. No prodromal symptoms were observed, as reported in other cases [18]. The infection was successfully treated with a DAIR and a second debridement after 10 days, which stands in contrast to the poor results described in the literature [22].

Osteoarticular infections due to *G. morbillorum* are rare (Table 1). Of all the reported cases, five were native septic arthritis [13,15], three of them were associated with an endovascular infection [16,18] or osteomyelitis [17], and two cases were associated with PJI (hip and elbow) [14,19]. To the best of our knowledge, the present case is the third case of PJI caused by *G. morbillorum*.

The two previous cases of periprosthetic infections, both chronic infections, were treated with two-stage prosthetic replacement. We concluded that the management of this case of acute hematogenous infection with DAIR was an option supported by the literature. According to the IDSA guidelines, patients should be considered for DAIR if the joint is less than 30 days old, or if the infectious symptoms last less than 21 days in the absence of a draining sinus tract [20]. Reported cases of septic arthritis in the native joint have been treated by means of arthroscopic lavage or aspiration and irrigation.

As endocarditis is the most common infection associated with *G. morbillorum,* a careful cardiac examination is always performed to rule it out in the case of a positive blood culture for *Gemella* or an infection in another location. In this case, it was negative.

Most of the reported cases involved predisposing or triggering factors of this infection. Those factors include endocarditis, gastrointestinal carcinoma, periodontitis and chronic corticosteroid use. This is the first case of osteoarticular infection caused by *Gemella* where the precipitating factor was advanced liver disease, specifically a previously undiagnosed decompensated cirrhosis due to alcoholic- and metabolic-associated fatty liver disease that was diagnosed after further study. This comorbidity conditioned the mechanism of the infection and the patient’s clinical course during admission. It is known that patients with liver cirrhosis have an increased risk of infection and in-hospital mortality or postoperative complications after surgery (renal, respiratory and infectious). Moreover, the hospitalization period and use of the intensive care unit are significantly greater among this population [19,20]. Intestinal bacterial translocation is a common cause of infections in cirrhotic patients, with the passage of viable bacteria or bacterial products from the intestinal lumen to the mesenteric lymph nodes and then to systemic circulation. This can lead to spontaneous bacterial peritonitis, which was ruled out in this case, or spontaneous bacteremia. In the case reported here, it turned out to be the latter, this being the final diagnosis proposed for this patient [23,24].

We used intravenous cefotaxime during hospital admission and oral amoxicillin for three months after hospital discharge. The isolated strain of Gram. Showed a susceptibility to all antibiotics: clindamycin (MIC ≤ 0.125 mg/L), cefotaxime (MIC ≤ 0.002 mg/L), penicillin (MIC ≤ 0.002 mg/L), vancomycin (MIC ≤ 0.25 mg/L) and rifampicin (MIC ≤ 0.002 mg/L). According to the literature, most cases of *Gemella* are susceptible to beta-lactams and vancomycin. Other antibiotics that may show activity against *G. morbillorum* are macrolides, levofloxacin and linezolid. They are inherently resistant to trimethoprim-sulfamethoxazole and streptomycin [25].

## 4. Conclusions

*G. morbillorum* osteoarticular infection is a rare but possibly underdiagnosed entity due to its difficult diagnosis. It should not be considered a contaminant if it is isolated in a synovial fluid analysis. We presented a case report of an acute hematogenous *G. morbillorum* infection successfully treated by means of DAIR and beta-lactam antibiotic therapy.

Since most patients who experience this infection present with predisposing factors, it is advisable to perform an echocardiography, gastroscopy, colonoscopy, and abdominopelvic CT scan, as well as an examination of the oropharynx and teeth, in order to identify an underlying condition. Spontaneous bacteremia of intestinal origin in patients with risk factors for immune dysfunction (e.g., liver cirrhosis) should also be considered in the differential diagnosis.

## Figures and Tables

**Table 1:** Summary of reported cases of septic joints caused by *Gemella morbillorum*.

Reference	Sex, Age	Joint Disease	Predisposing Factors	Surgery	Antibiotic Therapy
Von Essen et al., 1993 [19]	Female, 45	Total elbow arthroplasty	Chronic fistula	Two-stage revision arthroplasty	NS
Omran and Wood, 1993 [16]	Male, 48	Wrist	Poor dentition. Bacteriemia. Possible infected dialysis access graft	Wrist aspiration	Vancomycin 6 w
Van Dijk et al., 1999 [17]	Male, 42	Trochanter osteomyelitis and ipsilateral septic hip	Chronic corticoids	Open articular lavage	Penicillin G and clindamycin for 6 w. Oral clindamycin 3 w
Czarnecki et al., 2007 [18]	Male, 75	Knee	Bacteriemia. Endocarditis	Knee aspiration and irrigation	Ceftriaxone 6 w
Roche and Smyth, 2005 [15]	Male, 42	Knee	NS	Arthroscopic articular lavage	Penicillin G and flucloxacillin 6 d. Oral amoxicillin and oral flucloxacillin 5 flucloxacillin 5 w
Medina-Gens et al., 2007 [14]	Male, 41	Total hip arthroplasty	Periodontitis	Two-stage revision arthroplasty	Cloxacillin and gentamicin 4 d. Cefotaxime and gentamicin 11 d. Penicillin G 22 d Teicoplanin and rifampicin
Desmottes et al., 2018 [13]	Female, 90	Knee	Pseudogout	Arthroscopic articular lavage	Amoxicillin/clavulanic acid. Oral amoxicillin 6 w
Present case, 2021	Female, 60	Total hip arthroplasty	Cirrhosis	DAIR. Second DAIR after 10 d	Daptomycin, cloxacillin and ceftazidime 4 d. Cefotaxime and metronidazole 2 w Amoxicillin 4 w

## Data Availability

The datasets used in the current study are available from the corresponding author on reasonable request.
